# Modern Synthesis and Sintering Techniques of Calcium Copper Titanium Oxide (CaCu_3_Ti_4_O_12_) Ceramics and Its Current Trend in Prospective Applications: A Mini-Review

**DOI:** 10.3390/nano12183181

**Published:** 2022-09-13

**Authors:** Gecil Evangeline T., A. Raja Annamalai, T. Bonnisa Magdaline

**Affiliations:** 1 School of Mechanical Engineering, Vellore Institute of Technology, Vellore 632014, India; 2 Center for Nanoscience and Technology, Pondicherry University, Puducherry 605014, India; 3 Centre for Innovative Manufacturing Research, Vellore Institute of Technology, Vellore 632014, India

**Keywords:** Calcium Copper Titanium Oxide, synthesis routes, sintering techniques, dielectric properties, photocatalyst, zinc–air batteries

## Abstract

Calcium Copper Titanium Oxide (CaCu_3_Ti_4_O_12/_CCTO) has grasped massive attention for its colossal dielectric constant in high operating frequencies and wide temperature range. However, the synthesis and processing of CCTO directly influence the material’s properties, imparting the overall performance. Researchers have extensively probed into these downsides, but the need for a new and novel approach has been in high demand. Modern synthesis routes and advanced non-conventional sintering techniques have been employed to curb the drawbacks for better properties and performance. This review provides a short overview of the modern synthesis and sintering methods that utilize direct pulse current and electromagnetic waves to improve the material’s electrical, optical, and dielectric properties in the best ways possible. In addition, the current application of CCTO as a photocatalyst under visible light and CuO’s role in the efficient degradation of pollutants in replacement for other metal oxides has been reviewed. This research also provides a brief overview of using CCTO as a photoelectrode in zinc–air batteries (ZAB) to improve the Oxidation-reduction and evolution (ORR/OER) reactions.

## 1. Introduction

Calcium Copper Titanium Oxide (CaCu_3_Ti_4_O_12_/CCTO) ceramic came into the limelight after its huge dielectric constant (12,000) at room temperature was discovered in 2000. Another essential feature that fascinated the research community has been its unique cubic double-perovskite structure (AA’BO3), where Ca^2+^ is situated at A, Cu^2+^ at A’ space, and Ti^4+^ located at B site. The octahedral TiO_6_ forms a square planar structure produced by the JahnTeller distortion in Cu^2+^ [1,2,3,4]. It outperformed other perovskite oxides such as SrTiO_3_ and BaTiO_3_ in terms of holding a large dielectric constant without including additional copper into the structure. The initial processing procedures and the intrinsic barrier layer mechanism significantly contribute to the large dielectric constant of CaCu_3_Ti_4_O_12_. Perovskite structures, such as titanates, are well-known ferroelectric materials with exceptional dielectric properties. The term “internal barrier layer capacitors” (IBLC) refers to the phenomenon in which CCTO ceramics exhibit massive permittivities due to extrinsic polarization between semi-conductive and insulating grain boundaries. Internal barrier layer capacitors are formed as a result of this phenomenon. CCTO has been synthesized in numerous traditional ways, including solid state reactions, combustion, co-precipitation, and sol–gel techniques. Though these techniques yield homogeneous and desired outcomes, there has been an increased concern regarding unwanted residues, formation of secondary phases, and long processing time [5,6]. To address the shortcomings of the issues mentioned above, researchers have recently explored the latest methodologies that focus on rapid and environmentally friendly synthesis methods to yield high purity ceramic powders.

Similarly, traditional sintering methods where the samples are sintered in a muffle or box furnace at high operating temperatures have now been replaced with efficient advanced techniques that curtail the processing time and sintering temperature, achieving enhanced properties [7,8,9,10]. Based on the heating mode, applying pressure and current sintering techniques has been classified as Spark Plasma Sintering (SPS) or Pulsed Electric Current Sintering (PECS) and microwave sintering. In conventional sintering, the green compact or pellets are heated at a high operating temperature without any external pressure in a controlled atmosphere. Spark Plasma Sintering (SPS) employs a force field and electric field to generate products of desired density without coarsening. In total contrast, microwave sintering has been fostered by microwave irradiation at 2.45 GHz, generated using the magnetrons placed inside the chamber.

In addition, several researchers have probed the properties of CCTO materials by introducing dopants. The effect of Hf doping on the structural, dielectric, and optical properties of CCTO resulted in remarkable dielectric relaxation [11]. Wang et al. demonstrated the substitution of Nd at the Ca site in CaCu_3_Ti_4_O_12_ ceramics and the effects of Nd-substitution on the microstructures and dielectric characteristics [12]. Doping elements of interest to boost the electrical, nonlinear, and I-V properties has been the primary focus of researchers attempting to cater to ceramic fabrication in different application fields based on their properties [13,14,15,16]. This review provides a crisp and clear insight with special emphasis on modern novel synthesis routes such as microwave synthesis, molten salt synthesis, and microwave flash combustion methods to overcome the setbacks of traditional solid-state reaction and sol–gel techniques. This article also seeks to provide a brief overview of state-of-the-art sintering techniques, such as Spark Plasma Sintering (SPS) and Microwave Sintering (MWS), which use pressure and electromagnetic waves to process the feedstock on both the micro and nano-metric scale, resulting in the material of high quality and maximum performance. The most recent developments in using CCTO for harvesting and transforming energy sources have also been covered.

## 2. Advanced Techniques for Synthesis of CCTO Powder

### 2.1. Microwave Synthesis of Calcium Copper Titanium Oxide Powder 

**Figure 1:**
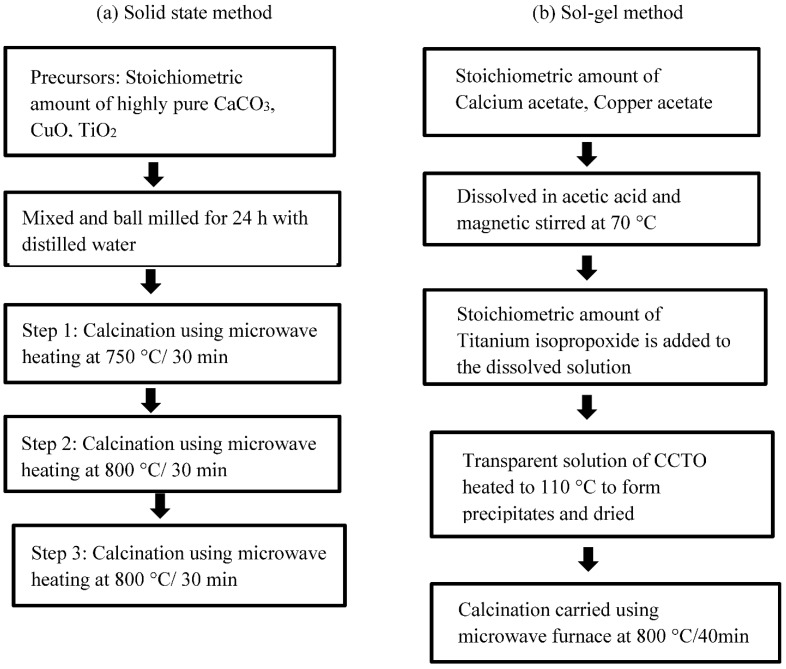
Flowchart of microwave synthesis of CCTO from (**a**) solid state precursors (**b**) Sol–gel precursors.

Many conventional methods, including solid-state reaction, sol–gel, sonochemical and self-propagating high-temperature synthesis, and co-precipitation approaches, have been used to produce calcium cotper titanium oxide [5,17,18,19,20]. Aside from a few limitations that include inhomogeneity, additional processing, and the need for high temperatures, these approaches nonetheless provide many advantages. Since ceramic synthesis necessitates a high temperature for the diffusion of atoms, the main focus in recent years has been reducing time and finding an efficient heating source. Because of microwave irradiation’s ability to speed up chemical processes and eliminate the need for numerous processing stages such as grinding, microwave synthesis has become a widely used approach [20,21]. Microwave heating was successfully used by Hongtao Yu et al. to manufacture CCTO powder in a single cubic phase. This method synthesized single-phase CCTO in less than two hours, requiring less energy than the standard synthesis route. The dielectric constant of the microwave-manufactured powder was higher than that of the conventional synthesized powder under the same sintering circumstances. P Thomas and his team attained the maximum dielectric constant via microwave synthesis using a modified home oven (2.45 GHz, 1.1 kW) in about 20 min [22]. Sol–gel precursors were utilized instead of solid precursors by Xin Ouyang. et al., who found that microwave heating produced 89.1 weight percent of CCTO in around 17 min, compared to conventional heating, which took 3 h to generate 87.6 weight percent of CCTO. Conventional heating has thus been beaten out by microwave heating because of the rapid heating rate and the smaller particle size of 3.826 μm. Since electromagnetic waves are converted into heat energy, they can be easily assimilated by the substance that expedites microwave synthesis, a potential method for calcination of precursor mixes. The SiC microwave-susceptors positioned around the alumina crucible aids to speed up the heating process by absorbing microwave energy and compensating for the temperature of the surrounding environment. As depicted in Figure 1a,b several precursors can be used in the microwave synthesis of the CCTO molecule [21,23,24,25].

### 2.2. Molten Salt Synthesis (MSS)

**Figure 2:**
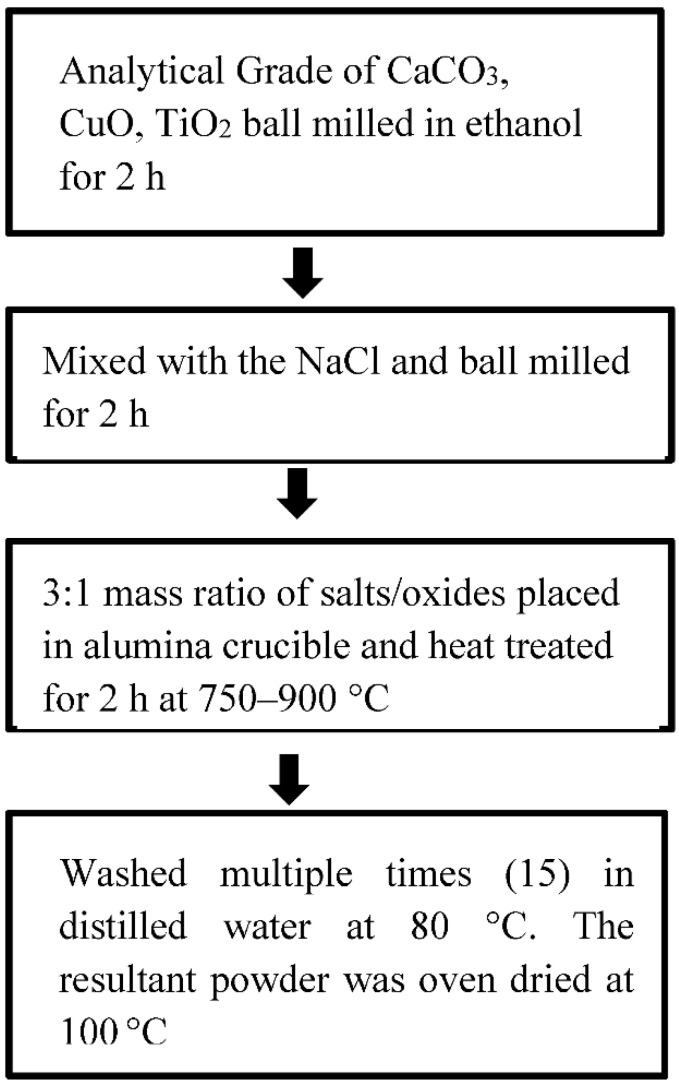
Flowchart of Molten Salt Synthesis.

The molten salt synthesis presented in Figure 2 impacts the crystallinity of CCTO particles at low temperature by heat treating stoichiometric amounts of CaCO_3_, CuO, and TiO_2_ in NaCl-KCl. CCTO powders were successfully synthesized using this MSS technique because the reacting ions’ enhanced diffusion rate in the salt medium reduced the requirement for high-temperature calcination. Kepi Chen et al. synthesized CCTO using a molten salt method where the precursors, namely CaCO_3_, CuO, and TiO_2_, were amalgamated into the molten salt mixture of NaCl/KCl and heated at 750–1000 °C for 1 to 16 h and thoroughly washed to eliminate the alkali metal salts. It has been manifest from the findings that the average particle size was maximized as the temperature inflated above 850 °C, and the holding time has influenced the morphology and grain size of the fabricated powder [26]. Furthermore, B S Prakash and his colleagues successfully produced nanocrystalline CCTO powders at 750 °C with 150 nm crystallites and a high dielectric constant post sintering as the KCl flux reduced the formation temperature [27]. The MSS route to CCTO powder fabrication at 850 °C produced a green body with a homogeneous microstructure and a mechanical strength of 9.27 MPa [28]. Wei Wan et al. reported improved dielectric performance of CCTO powder, synthesized at 800 °C using NaCl flux. They found no secondary traces of CuO and CaTiO_3_, which are normally predominant at low synthesis temperatures (750 °C). Though the MSS method has advantages over low-temperature synthesis and molten salt heterogeneity, the salt removal process has been significantly repressed [29].

### 2.3. Microwave Flash Combustion Method

The polycrystalline CCTO ceramic nanopowder (50 to 70 nm) was first synthesized using microwave flash combustion. In this novel technique, the aqueous solution is subjected to microwave irradiation in a mixture containing oxidizers and fuel. CCTO residual powder was calcined at 800 and 900 °C/5h to eliminate the as-obtained powder’s secondary residues of CaTiO_3_, TiO_2_, and CuO. Because of this, the dielectric constant rose to 20,000 with a tan loss of 0.51 at 100 Hz. The sintered pellets’ improved dielectric characteristics have been attributed to their nanoscale morphology [30].

**Figure 3:**
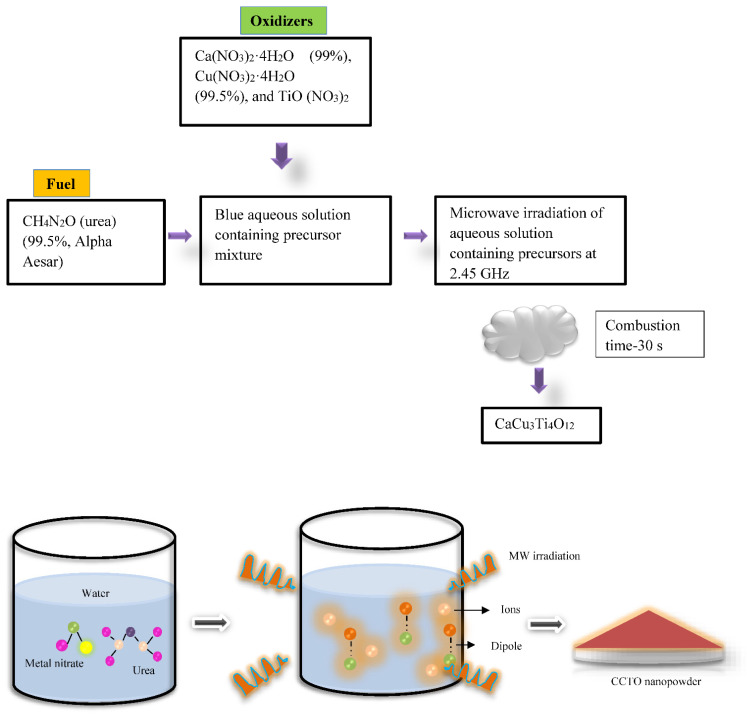
Schematic representation of microwave flash combustion method for production of CCTO powder (50–70 nm).

This method has been unique from other synthesis routes since the reaction takes place at the molecular level, reducing the processing time to a greater extent. The particles have been bombarded with electromagnetic waves that improve the kinetics response, resulting in ahigh purity nanopowder (50–70 nm) within a short span, as depicted in Figure 3 [31].

**Figure 4:**
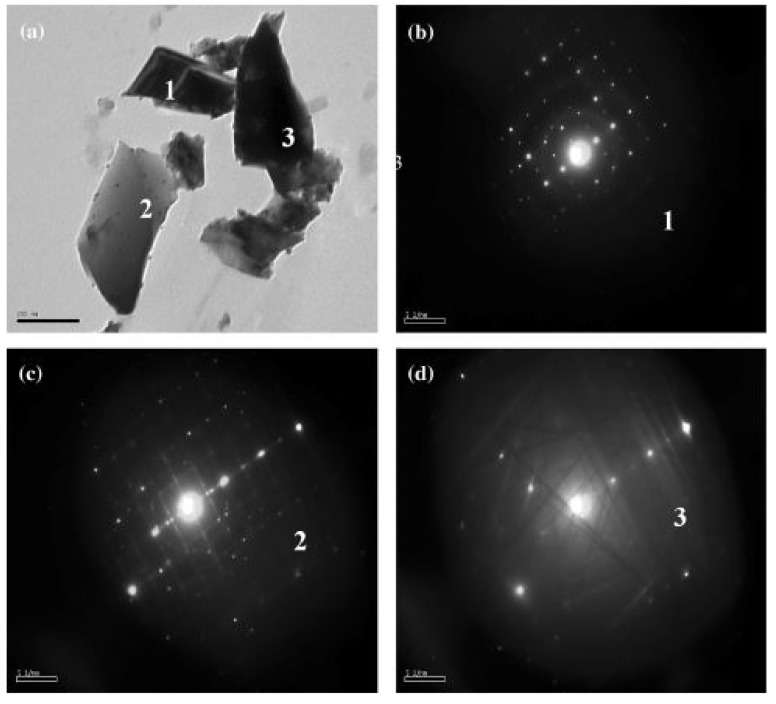
TEM images of (**a**–**d**) selected area electron diffraction (SAED) pattern of CCTO [36].

In recent years, new and novel modified eco-friendly, cost-efficient and quick synthesis techniques have been considered to overcome the limitations of contamination and environmental issues [32,33]. In addition, CCTO powder with a particle size of 4.78 nm and a single cubic crystal structure has been successfully manufactured by modifying the general synthesis procedure. This results in an output beneficial for energy storage applications [34]. The highly orientated single-cubic crystal structure [35] has been revealed by the presence of Kikuchi lines in the selected area electron diffraction (SAED) pattern obtained using HRTEM. This pattern is displayed in Figure 4 [36]. The consolidated outline of all modern synthesis methods discussed above has been briefed in Table 1.

## 3. Conventional Sintering

### 3.1. Grain Growth Mechanism

**Figure 5:**
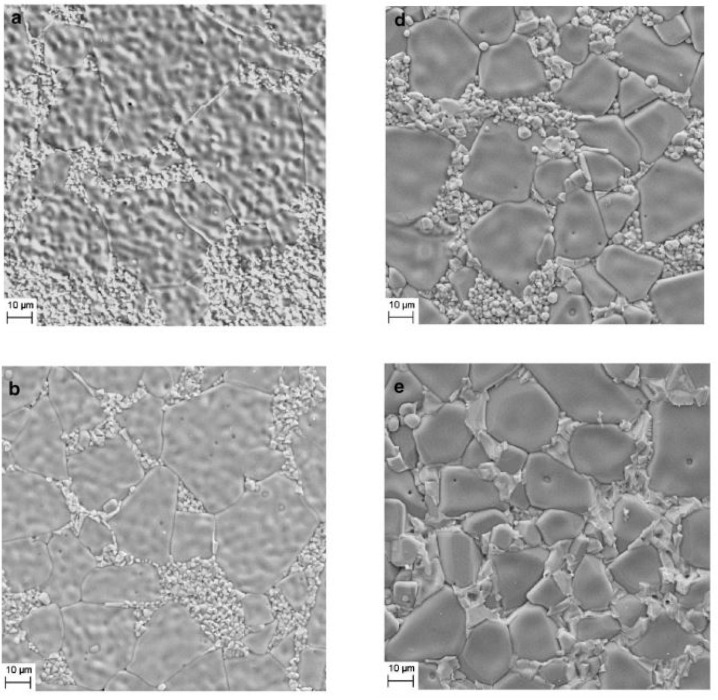

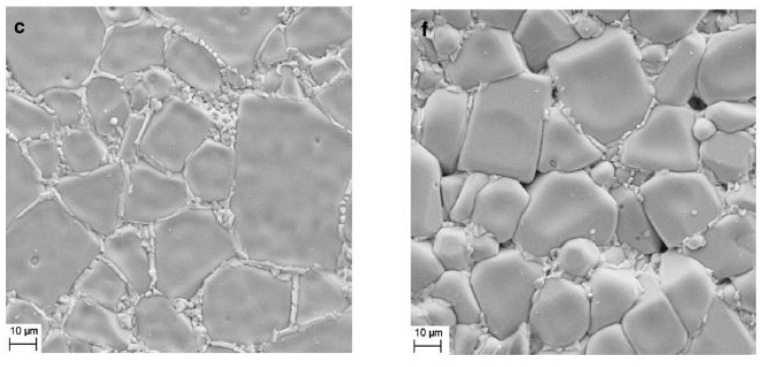
FE SEM images of samples sintered at (**a**) 1050 °C/2 h, (**b**) 1050 °C/4 h, (**c**) 1050 °C/12 h, (**d**) 1100 °C/2 h, (**e**) 1100 °C/4 h, (**f**) 1100 °C/12 h [9].

**Figure 6:**
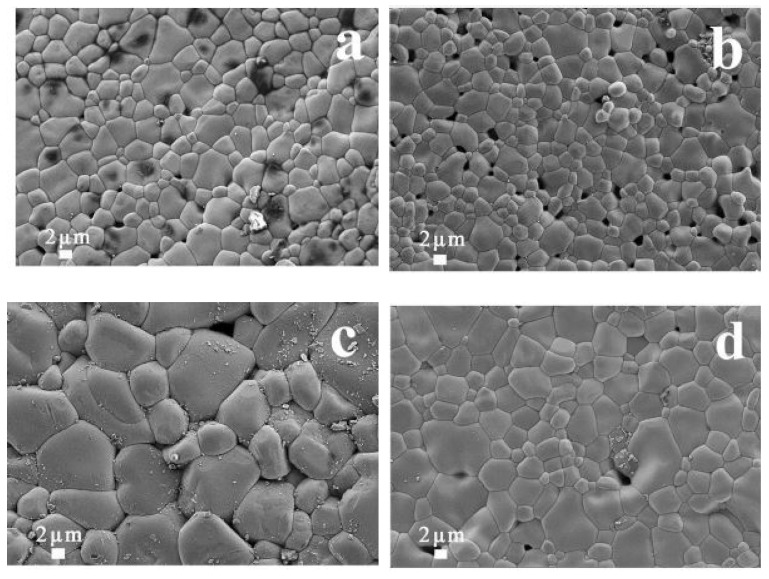

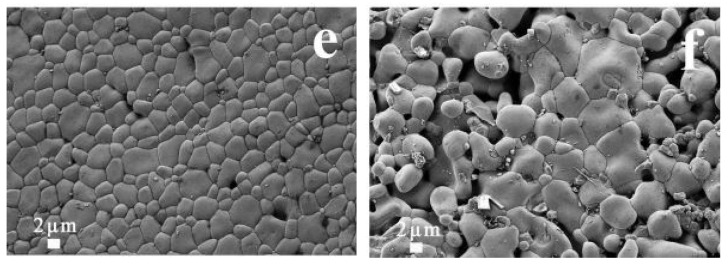
SEM images of CCTO powders obtained by calcination at (**a**) 700 °C, (**b**) 750 °C, (**c**) 800 °C, (**d**) 850 °C, (**e)** 900 °C and (**f**) 1000 °C by Wang et al. [40].

**Figure 7:**
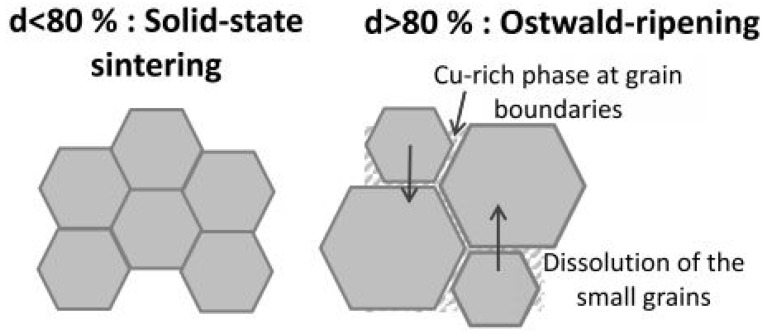
Proposed scheme of grain growth mechanism reported by Guillaume Riquet et al. [39].

Numerous researchers developed innovative, cutting-edge methods and procedures to address the drawbacks of having a high dielectric constant usually accompanied by a significant dielectric loss. Even if the dielectric constants were susceptible to processing inputs, the dielectric loss has always been linked to increased frequency. This is because dielectric loss is always proportional to the square of the frequency. A modified solid-state reaction technique that Sanjesh Babu and his colleagues developed incorporates further particle size reduction into the overall process. The starting mixture was reduced to finer particles using a sieve of a particular size after milling and heating at 1100 °C in a high-grade alumina crucible. This study reported an increase in dielectric constant and dielectric loss as the temperature increased, (30–250 °C) and vice versa for an increase in frequency (100 Hz–100^4^ Hz). Subsequently, this mechanism has been associated with a lack of interfacial polarization at high frequencies [37]. The microstructure of CaCu_3−x_Ni_x_Ti_4_O_12_ (CCNTO) manifested large and abnormal grains at (x > 0.1) for 24 h, as delineated in Figure 5 and Figure 6 which reveal the effect of dwell time and calcination temperature on the intrinsic properties of CCTO. Guillaume Riquet et al. observed the sintering mechanism over the evolution of the microstructure of the CCTO ceramics, as illustrated in Figure 7. The densification process was probed using the sintering trajectory (i.e., grain size vs. density) at a temperature starting from 1000 to 1100 °C for a dwell time of 2 h. It has been noted that the initial densification (60% to 80% theoretical density) was due to the grain boundary diffusion, where the grain size almost remained constant.

Similarly, for higher densities more significant than 80%, the grain size growth increased rapidly because of the Cu-rich liquid phase at the grain boundaries [29,38]. The Cu-rich liquid phase expedited this phenomenon, known as Oswald Ripening (a dissolution–diffusion–precipitation mechanism). However, this Cu-rich phase has to be further validated and examined using transmission electron microscopy [39].

### 3.2. Effect of Doping on Electrical and Microstructural Properties

Doping different elemental compounds have reinforced the electrical and microstructural properties of CCTO ceramics. The CCTO ceramics, doped with Nickel (Ni) concentration (x = 0, 0.05, 0.075, 0.1, 0.15, and 0.2) and prepared using the solid-state route, have been conventionally sintered in air at 1100 °C with a dwell time of 6, 12, and 24 h. The average grain size of the ceramics has been induced by the nickel concentration and dwell time. It has been conspicuous that the grain growth progressed with an increase in sintering time and Ni concentration, but the average grain size was substantially reduced [41]. In another work, Nickel (Ni)-doped CCTO exhibited a shallow dielectric loss of 0.07 and a dielectric constant of 6 × 10^3^ MHz [42]. Similarly, cobalt addition to pure ceramics has decreased the dielectric loss at low and middle frequencies. The CaCu_3−x_Co_x_Ti_4_O_12_ (CCCTO, x = 0, 0.2, 0.4 and 0.6) has been synthesized using semi-wet route and sintered at 1050 °C for 15 h in air. This investigation concluded that the dielectric constant was exacerbated by the cobalt concentration rise due to substituting Cu^2+^ with Co^2+^. Furthermore, the compositions that possessed a composition (x = 0.6) and (x = 0.4) were found suitable for application in wide temperatures (300–600 K) and frequencies up to 20 kHz with low dielectric loss. The dual combination of enhanced dielectric constant (1.7 × 10^4^ < ε > 6 × 10^4^) and reduced dielectric loss (0.87 to 0.15) was achieved by reduction of the CuO-rich phase by the formation of CaTiO_3_ and Co_2_TiO_4_ phases, which elevated the resistance at the grain boundaries [43] as mentioned in Table 2. The dielectric properties of CaCu_3_Ti_4_O_12_ were also tuned by substituting 1 mol% Bi and Al, which improved the breakdown field from 2088 V/cm to 7839 V/cm due to the resistivity of grains and enhanced Schottky barrier height. In the current research study, substitution has been proven to boost the effective permittivity to 71,000 with a loss of 0.047 at 1 kHz by co-doping with elements like aluminum and fluorine [44]. Significantly, the substitution of new dopants like In^3+^/Ta^5+^ in the vacant Cu and Ti sites has reduced the dielectric loss, which has never been reported in earlier works [45]. Ravikiran et al. examined the optical gap of CCTO with Hafnium (Hf) which had an inverse effect on the dielectric properties. Substitution with Sr^2+^, Ge^4+^ in the Ti sites recorded an enormously improved dielectric response, which elucidated a highest dielectric permittivity value of 69,889 and the comparatively lowest dielectric loss of 0.03 [46]. Researchers like Jakkree Boonlakhorn have been extensively working on doping various elements to investigate the electrical and dielectric components of the new CCTO ceramics. This includes doping of Cd^2+^, F^−^ into the binary system of CCTO/CTO to enhance the dielectric permittivity twice that of pure CCTO and reduce the loss tangent of 0.03 [47]. The addition of rare earth elements to the CCTO structure gains prospective interest owing to the colossal dielectric constant at a high operating frequency range. The dielectric loss was reduced to a very minimal value [48,49,50,51,52].

## 4. Spark Plasma Sintering (SPS)

### 4.1. Significance of Pressure and Pulsed Current in Rapid Densification

Spark Plasma Sintering (SPS) or Electric Discharge Sintering (EDS) is a rapid sintering technique extensively used for solid consolidation of powder samples under applied uniaxial pressure, pulsed and high DC, and low voltage. This method has seen widespread use to achieve desirable densification of CCTO samples because of its rapid heating rate, short sintering time, and improved properties such as enhanced mechanical properties, high permittivity, and reduction of impurities along the grain boundaries. This is demonstrated by the fact that rapid sintering can be accomplished by applying pressure and pulse current, which is why this method has been fruitful.

**Figure 8:**
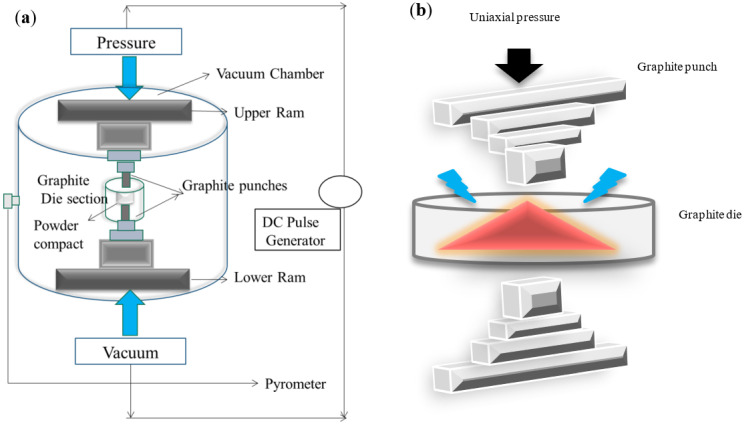
Schematic representation of (**a**) Spark Plasma Sintering (**b**) Mechanism of SPS.

The electric discharge and the external pressure will cause the powder particles to become activated, which will then promote diffusion bonding between the particles, resulting in a high density [53]. As depicted in Figure 8a,b, the mechanism involves densification of the particles by uniaxial pressing and simultaneously heating up of the die and punches by their resistance. The reduction in porosity has been achieved by restricting particles’ movement by intensifying lattice and boundary diffusion. Most significantly, SPS has been preferred over conventional sintering to avoid coarsening grains, which is common in a traditional heating mode [54,55]. Despite this, the processing variables must be channelized into acquiring desired features of CCTO ceramics [56,57].

### 4.2. Effect of Dwell and Annealing Temperature

CaCu_3_Ti_4_O_12_ ceramics were exposed to various dwell temperatures of 850, 900, 915, and 930 °C, and their impact on the microstructure and the nature of grain distribution was investigated by E. de Carvalho et al. The refined powder was compacted and sintered using spark plasma sintering for a minimum of 2 min at an applied pressure of 60 MPa. As the dwell temperature increased, the densification ramped up with the increase in the mean grain size above 850 °C. As the grain size reduced, the dielectric permittivity of the sintered samples also dropped. All the sintered samples exhibited high permittivity, as mentioned in Table 3. However, below 915 °C, low densities were reported, and the ceramics’ nature turned brittle at 930 °C due to carbon contamination [58].

Xue-Feng Ruan et al. experimented using the SPS technique and examined the dielectric properties of CCTO ceramics by varying the annealing temperature (500, 600, 700, 800 and 900 °C). In a graphite die, the samples were sintered at 980 °C for 5 min under a pressure of 57 MPa. It has been displayed from these studies that the dielectric constant and loss decreased as the annealing temperature increased. This has been due to the oxygen vacancies that reduced the lattice constant and holes during annealing treatment. Similarly, the residual carbon reduced by annealing in a muffle furnace for 700 °C/10 h has elevated the electrical properties by reducing the thickness of the grain boundaries [59].

### 4.3. Influence of Sintering Temperature on the Microstructure and Dielectric Properties

The reduction of CCTO ceramic precursors to nanopowders using mechanochemical synthesis and subsequent sintering using SPS at temperatures of 800, 900, 975, and 1050 degrees Celsius for 10 min at pressures of less than 60 megapascals showed a significant increase in giant dielectric constant (GDC) due to the contribution of IBLC by dense grains. The grain size rose as the temperature of the sintering process did, giving results superior to those produced by standard methods of multiple-stage sintering [60]. Furthermore, microstructural changes were discovered to effectively modify the nonlinear current-voltage properties of CCTO in a short period (5 min) and were used for applications such as varistor [61]. Pu Mao and colleagues reported a high dielectric constant with smaller grains that had not previously been observed using the conventional method. Grain growth in SPS increased as the sintering temperature increased from 800 to 900 °C.

However, using pulse current inhibited the grain growth, resulting in grains of smaller size. Significantly, CCTO sintered at 900 °C achieved a high dielectric constant, maximum nonlinear coefficient (9.07), and an increased breakdown of 3256.59 V/cm. The ceramic samples prepared by the SPS method played a crucial role in energy storage application, owing to their intrinsic properties. These came from mechanical and dielectric properties improved by the absence of porosity and impurities along the grain boundaries due to electric field diffusion combined with thermal diffusion [20].

Similarly, recent work using SPS at 900 and 950 °C disclosed a low dielectric loss of 0.02 at 10 kHz with good nonlinear characteristics [62]. The corresponding dielectric and dielectric loss for various temperatures have been enlisted in Table 3. The grain size and evolution of the microstructure at different temperature ranges have been represented in Figure 7. Adding dopants such as Zn and Al, such as Zn and Al, improved the nonlinear properties with a reduced dielectric loss at 1 kHz. Secondly, the ceramics’ resistivity intensified with an increase in Al doping due to substituting Ti^4+^ ions with Al^3+^ ions.

**Table 3:** Grain size, the corresponding dielectric constant, and loss values for samples sintered from 800 to 900 °C [20].

Samples	Grain Size	ε′	Tan δ
SPS-800	2.23 (±1.56) μm	7.68 × 10^3^	0.119
SPS-850	3.66 (±2.24) μm	1.07 × 10^4^	0.141
SPS-900	4.68 (±2.45) μm	1.58 × 10^4^	0.490

## 5. Microwave Sintering (MWS)

### 5.1. Fundamentals of Microwave Sintering

The microwave sintering approach has been adapted because of uniform heating by volumetric absorption of MW radiation and rapid heating rates. In microwave heating, the electrical energy is converted to heat through the microwave (300 MHz to 300 GHz) interaction with the sample. Maxwell’s equation has been applied to estimate the microwave power absorbed, since the absorbed MW power is proportional to the electric field distribution of the sample [63,64]. The power absorbed per unit volume, P (W m^−3^), is expressed, as indicated in Equation (1).

P = σ |E|^2^ = 2 π f ε_0_ ε′_r_ tanδ |E|^2^ (1)

where E (V m^−1^) is the magnitude of the internal field,

σ is the total effective conductivity (S m^−1^),

f is the frequency (2.45 GHz),

ε_0_ is the permittivity of free space (ε_0_ = 8.86 × 10^−12^ F m^−1^),

ε′_r_ is the relative dielectric constant, and tan δ is the loss tangent [65].

**Figure 9:**
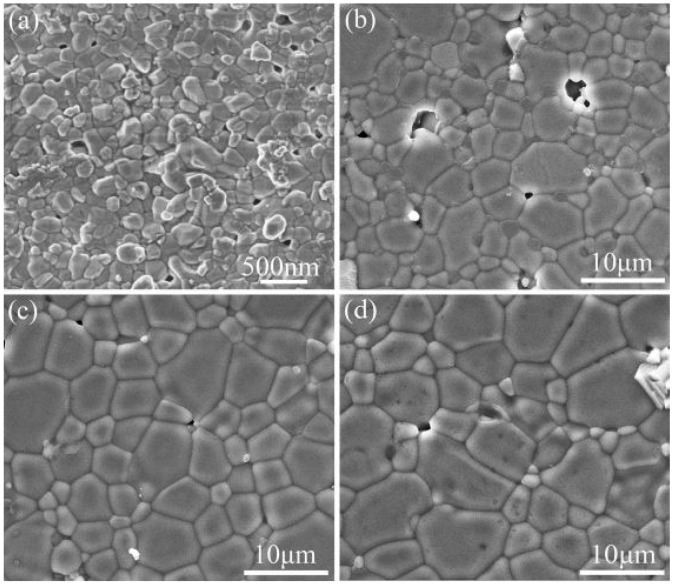
SEM images of (**a**) CCTO powders and CCTO samples sintered by SPS at (**b**) 800 °C, (**c**) 850 °C, (**d**) 900 °C [20].

**Figure 10:**
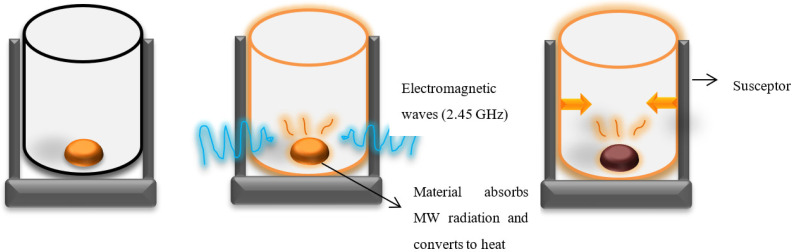
Schematic representation of Microwave sintering.

There are many potentially beneficial facets of the microwave sintering technology, some of the most promising of which include the promotion of high density, the inverse distribution of porosity even at a low processing temperature, and grain size close to the initial stage before processing. When it comes to ceramics, the absorption changes depending on how the rotation of bi-vacancy dipoles causes the heating to be induced; on the other hand, the heating has not simply been attributed to the electric field; instead, the interaction of particles and the phenomenon of the mass transport through the non-thermal microwave effect also plays a significant role in the heating of the sample [66,67]. Similarly, microwave sintering has also been utilized in the production of electroceramics to make use of the material’s fine microstructure, increased mechanical and dielectric capabilities, and high density (97 percent theoretical density). The processing of ceramic materials must take place at high dwell temperatures, as is general knowledge; this entails a large expenditure of time and energy. The new technology of microwaves, which is not utilized in regular processing, comes to the rescue as an alternate technique to address the drawbacks of standard processing [68]. Because of this, there is a pressing need to make high-quality ceramics [69] in a shorter period due to the desire for a big dielectric constant coupled with a low dielectric loss in CCTO. This may be explained by the fact that there is a desire for a shorter production time. The microwave sintering process, depicted in Figure 9, enables the internal heating of the material to be accelerated by the molecular interaction of the particles with the electromagnetic waves. This follows Maxwell’s derivation of the electromagnetic principle, which states that this can happen. Once the green compacts have been sintered, they will have mechanical properties, a net shape that is close to the required product, a density that is close to the theoretical density, and the single-phase pure phase that is intended together with controlled grain growth [70,71]. The mechanism of microwave heating inverse of temperature profile has been illustrated in Figure 10.

### 5.2. Comparison of Microwave Sintering over Conventional Sintering

**Figure 11:**
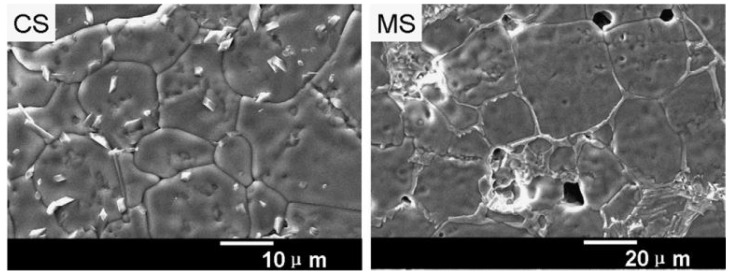
SEM images of sintered pellets from MS and CS powder [23].

**Figure 12:**
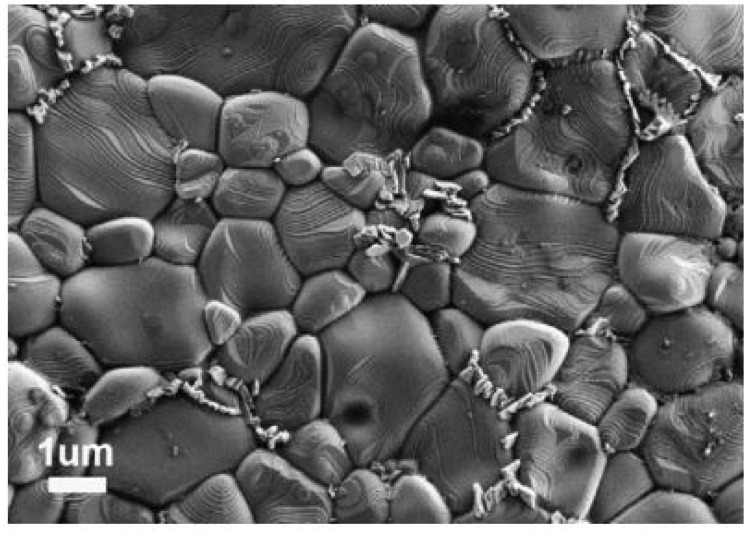
FESEM surface images of MW-sintered CCTO for 120 min that was pre-sintered at 1000 °C/10 h using a conventional furnace [73].

Hongtao Yu et al. made a comparative study between conventional and microwave-processed CCTO powders. The precursor mixtures were heated in the microwave (2.45 GHz, 1600 W) and conventional electric furnace (1000 °C). The principle behind microwave heating was the internal heating that occurred due to heat absorption from the crucible, surrounded by susceptors like SiC. The microwave synthesis process successfully generated a single-phase CCTO in 2 h, when a conventional technique required 24 h. After synthesis, the dielectric properties of microwaves and traditional synthesized powders sintered at 1100 °C for 3 h were measured to be (~21,400 and ~10,240) at 1 kHz. In this work, the morphology indicated in Figure 11 connoted that the sintered pellets of microwave-synthesized (MS) powders possessed large grains compared to conventionally synthesized (CS) powder. However, the porosities present in the sintered samples of MS powder yielded higher dielectric losses than CS powder. The porosity reduction has been addressed by elevating the calcination time in the current study for upcoming years [72]. In addition, the microwave-sintered samples fabricated were highly dense with uniform grain size, as Sabar D. Hutagalung et al. reported. It has been divulged in Figure 12 that the output product had high density with relatively high permittivity (ε_r_ = 2800), and low dielectric loss of 0.08 post-microwave sintering (120 min) and pre-sintered at 1000 °C/10 h in a conventional furnace [73].

### 5.3. Effect of MWS on Dielectric Properties

M A Ramirez et al. demonstrated conventional and microwave sintering to analyze the non-ohmic and dielectric properties of CaCu_3_Ti_4_O_12_/CaTiO_3_. In this investigation, the calcinated powders were sintered for three hours at 1100 oC in a conventional furnace and thirty minutes at 1050 oC in a microwave furnace. A heating rate of 5 °Cmin^−1^ was used for traditional heating and 230 °Cmin^−1^ for microwave radiation. In the end, it was found that the high-density ceramics produced by the MW technique gave the CCTO/CTO composites a nonlinear coefficient value of 65 for microwave-sintered samples and 42 for conventionally sintered samples. At high frequencies, the relative dielectric permittivity of CS samples (2960) is higher than that of MW-sintered samples (2100) at 1 kHz. In this way, microwave sintering ceramics can be used to produce varistors [74]. Microwave sintering at 1000 °C for 60 min resulted in maximum dielectric values of 11,000–7700 over the 100 Hz–100 kHz range for CCTO powder. In contrast to conventional methods, the dielectric loss was significantly reduced with increased frequency.

Ranjit Kumar and his colleagues derived a relation between the microstructure and the dielectric properties of the ceramics that were microwave-sintered over a temperature range of 1000, 1025, 1050, and 1075 °C for 15 min at 50 °C min^−1^ heating rate. Increased sintering temperature-increased grain growth and escalated the dielectric constant (∼6800) with a dielectric loss of 0.12 at 10 Hz. This low dielectric loss has been ascribed to the grain and grain boundaries [75]. Current research studies on the dielectric properties of CCTO ceramics [16] by adding rare earth elements like erbium (Er) fostered using microwave sintering have improved the dielectric characteristics at high frequencies. The CaCu_3_Ti_(4−x)_Er_x_O_12_ (x = 0, 0.02, 0.1, 0.2, 0.5 and 1.0) ceramics were processed via sol–gel method and then sintered in a microwave furnace at 1000 °C for 10 min. Significantly, the substitution of 0.5 mol% Er in Ti sites has improved the dielectric constant (∼11,700) of CCTO in a concise duration of 10 min [76]. Accordingly, the CCTO ceramic fabricated via MWS yielded a dielectric constant > 10^4^ and a very low dielectric loss (0.09) at 100 kHz [77]. Further addition of La to pure CCTO, which demonstrated better dielectric properties at medium and high frequencies [78]. The dielectric constant and loss for different concentrations of Er doping have been enlisted in Table 4. Though microwave technology seems to be the best alternative for processing ceramics, this field has to be explored in the near future.

## 6. Applications

In the current energy scenario, energy storage and harvesting rely on oxygen electrochemistry via fuel cells, and metal-air batteries have been a promising option for clean energy storage. [7,8,9,10,11,12,13,14,15,16,17,18,19,20,21,22,23,24,25,26,27,28,29,30,31,32,33,34,35,36,37,38,39,40,41,42,43,44,45,46,47,48,49,50,51,52,53,54,55,56,57,58,59,60,61,62,63,64,65,66,67,68,69,70,71,72,73,74,75,76,77,78,79,80,81]. It is cost-effective, abundant, high stability, and less toxic in alkaline and aqueous medium [82,83]. The platinum and platinum alloys have proven to be the most effective ORR catalysts that exhibit weak OER performance. The CaTiO_3_ exists as orthorhombic and cubic crystal structures on the calcination temperature. At temperatures below 1380 K, they exist as an orthorhombic structure with space group P_bnm_. Above 1580 degrees Kelvin, the orthorhombic structure shows evidence of changing into a “tetragonal” structure with space group I4/mcm and also displays a cubic structure with space group Pm3m. Both of these structures are possible transformations of the orthorhombic structure.

In this context, the CaCu3Ti4O12 (CCTO) ceramic, which is a 1:3 A-site ordered perovskite (A′A″3B4O12), has been renowned for its enormous permittivity, which has gained attention extensively in the recent years [84,85]. Metacomposites and metamaterials with negative permittivity or permeability opened a new avenue to realize extraordinary electromagnetic (EM) properties, which can be utilized for various applications, including invisibility, sensors, microwave absorbers, and electromagnetic shielding, among others. The metamaterials apprehended negative parameters by constructing the periodical unit cells that limited their applications owing to the narrow bandwidth and tedious preparation process. On the other hand, ferroelectric crystals of ABO_3_ type perovskite structure have been employed in various applications such as capacitors, electrical and magnetic components, and infrared sensors. Several attempts have been made to synthesize materials with a dielectric constant in broad ranges of temperatures and frequencies. Other applications, including the synthetic rock used for storing nuclear waste and for various catalytic applications like partial oxidation of light hydrocarbons [86,87,88]. Luangchuang et al. reported improved flexible dielectric materials using polar NBR as the matrix owing to its high dielectric constant, processability, and thermal resistance. CCTO was considered a good ceramic filler because of its excellent dielectric constant and nontoxicity [89]. Because of their photocatalytic behavior in visible light for pollution degradation, CCTOs have potential applications beyond their high dielectric permittivity and thermal stability. Besides, CCTO can be exploited mainly for gas sensing applications as thin films over the substrate, which has been a suitable replacement for semiconducting metal oxide sensors [90,91].

### 6.1. Paper-Based Zinc–Air Batteries

**Figure 13:**
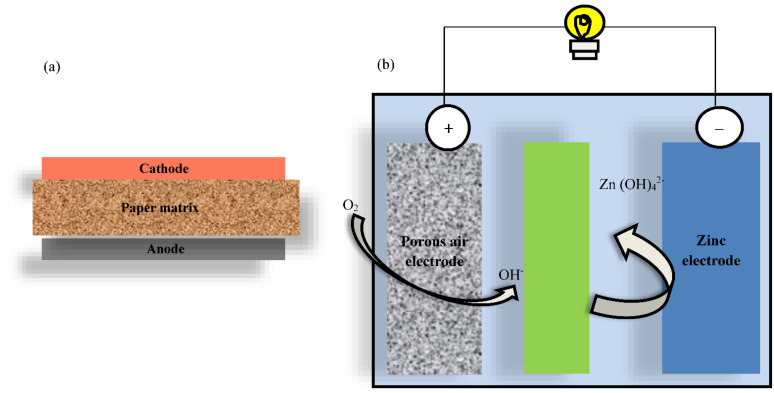
Schematic representation of (**a**) paper-based battery and (**b**) Zinc–air battery.

CCTO incorporated in paper-based x-zinc–air batteries showcased in Figure 13 exhibited improved ORR and OER, resulting in better cyclic stability and increased battery power density. In recent years, interest in paper-based batteries has grown due to their versatility, low cost, and ease of disposal. Glass and silicon substrates have now been substituted with paper-based batteries, which are more environment-friendly. Paper is biodegradable and can be dissolved at the end of its product life, which is why this paper-based substrate has been chosen. Paper can also be used as a free-flowing medium for ion transport. The outstanding performance and energy density of paper-based lithium-ion batteries have inspired researchers to try out other battery technologies in paper-based devices, including supercapacitors, mechanical nanogenerators, and electrochemical batteries [92,93,94]. As previously described, there are numerous advantages and benefits of using paper as a substrate for a battery. Much attention has been paid to zinc–air batteries in developing portable electric cars, electronic devices, and grid storage.

Because of its power density (1353 W h kg^−1^), zinc–air batteries outperform lithium-ion batteries over five times. In alkaline and aqueous media, it has lower toxicity and is more cost-effective, abundant, and stable than most sustainable energy storage options. While ZABs have a lot of potential, some obstacles, such as a lack of reversibility, low energy efficiency, and low output power density, make using ZABs in real-world applications a big challenge. The ORR–OER reactions regulate various electrochemical processes in energy conversion and storage devices. In terms of ORR catalysts, platinum and platinum alloys are the most effective. Due to byproduct generation and delayed reactions at the cathode reducing efficiency, they appear promising at first glance.

CCTO has been used as an electrocatalyst for the ORR/OER reactions as a remedy to the aforementioned difficulty. Because of their high specific capacity, structural flexibility, and low cost, perovskites are currently chosen in ZAB applications [95]. This is the first time CCTO has been used for zinc–air batteries (ZAB), and the specific capacity of 614 mAh g^−1^ was achieved [96,97]. Compared to iridium and rhodium oxide, which had poor ORR behavior in ZAB [98], CCTO exhibited excellent performance. It has been determined that the oxygen vacancy promoted the absorption of oxygen, which in turn increased the oxidation-reduction reaction’s activity. In addition, the increased electrocatalytic activity of the extra Cu and Ti aided the oxygen processes. The battery’s cyclic stability and high power density were raised due to the ZAB’s improved ORR and OER combined with CCTO [99].

### 6.2. Energy Storage and Energy Conversion

In contrast to batteries, which use electrochemical reactions to store energy, capacitors use static energy. At working temperatures and frequencies, materials with sizeable dielectric permittivity can be found in supercapacitors. Microelectronic devices of outstanding quality and physical qualities are in high demand. The movement of ions and electrons provides the driving force for batteries. An equation has been used to estimate stored energy in capacitors, driven by a parallel plate design (2) [100].

E = (1/2) CV^2^ (2)

where,

E—Energy stored,

C—Capacitance,

V—Voltage applied.

The breakdown electric fields (4400 V/cm) and nonlinear coefficients (6.3) of CCTO, due to multiple energy gaps, produced at the grain boundaries produce excellent discharge current and energy decay response, which attracts the attention of the capacitor fabrication industry [101]. In energy conversion, CCTO has employed third-generation visible light photocatalysts with a photocurrent density of 0.96 mA/cm^2^. The photocatalytic behavior of CCTO plays a vital role in photoelectrochemical cells (PECs). Novel CCTO under visible light intensity (100 mW cm^−2^) incorporated as a photoanode in the photoelectrochemical cell has demonstrated high efficacy for solar energy conversion. Sara Kawrani and their team prepared CCTO with graphene oxide nanosheets. They observed 50% hydrogen production along with enhanced photocurrent generation due to the segregation of CuO along the grain boundaries [102]. Henceforth, CCTO acts as a photoactive electrode under visible light in photoelectrochemical cell (PEC) and aids in energy conversion owing to the double-perovskite structure and entrapment of electrons by CuO present along the grain boundaries [103]. The photocatalytic behavior of CCTO under visible light having narrow band gaps (2.21 and 1.39 eV) shows a promising application for water splitting and degradation of pollutants subjected to light and not specific to UV radiation like other metal oxide materials [104,105,106,107].

## 7. Conclusions

This review highlights new synthesis approaches for the synthesis of CCTO powders. These new approaches include molten salt synthesis, microwave synthesis, and microwave combustion routes. It was discovered that these methods are an adequate replacement for the conventionally utilized paths, which require a significant amount of time and result in higher production costs. When subjected to microwave combustion, a powder of nanometric pure grade is produced. When subjected to sintering, this powder has excellent electrical and dielectric properties. Spark plasma and microwave sintering are two examples of the more modern techniques that have supplanted the more time-honored, traditional method of sintering, which had been in use for several decades. Because of these more recent techniques, rapid fabrication at a lower processing temperature became something that commercial producers show greater inclination to. In addition, the most recent advancement in processing methods uses a direct pulsed current in conjunction with pressure.

In contrast, the final product demonstrates uniformity in grain size distribution after being subjected to microwave irradiation, which is desirable for applications involving capacitors. This study also reveals a wide range of processing variables that significantly impacted the dielectric response of pure and co-doped CCTO ceramics. Based on the method of fabrication, CCTO ceramics have the potential to find a market in the production of microelectronic devices and applications involving energy storage. However, the processing variables should be investigated in great detail, and an alternative for novel methods should also be devised to eliminate significant shortcomings in terms of cost and time. The photocatalytic behavior of CCTO has also been a future application for water treatment in the degradation of pollutants due to narrow band gaps under visible light. This application has yet to be fully explored, but it has the potential to be in the near future.

## Figures and Tables

**Table 1:** Process, procedures, advantages, and drawbacks of different synthesis routes.

Synthesis Route	Precursors	Processing Temperature/Time	Advantages	Disadvantages
Microwave synthesis	Solid state	950 °C/20 min	Powders synthesized via this technique yielded a better dielectric constant and subsequently low dielectric at high frequency than the conventional synthesis route.	- [22]
	Solid state	800 °C/30 min	Single phase cubic CCTO was obtained with less energy consumption and short processing time.	Repeated calcination is to be ensured to remove secondary traces [23].
	Sol–gel	950 °C/17 min	89.1 wt% was fabricated in a shorter duration with particles measuring 3.826 microns. The resultant powder demonstrated excellent dielectric performance along with low dielectric loss.	- [24]
	Sol–gel	800 °C/40 min	CCTO powder of desirable grade has been manufactured at low processing temperatures.	- [25]
Molten Salt Synthesis	CaCO_3_, CuO and TiO_2_ NaCl/KCl salts	750–1000 °C/ 1–16 h	High influence of holding time on the particle size.	High temperature and processing time [26].
	CaCO_3_, CuO and TiO_2_ 0 to 20 mol% KCl	750 °C/10 h	Phase pure CCTO of size ~150 nm has been obtained over conventional routes requiring high temperatures of 1000 °C. KCl flux played a prominent role in reducing the formation temperature of CCTO.	- [27]
	CaCO_3_ (≥99.0%), CuO (≥99.0%) and TiO_2_ (≥99.0%) NaCl flux	750–850 °C/2 h	Green compact of high homogeneity and mechanical strength. Gel casting exhibited lower loss (0.2) and a higher dielectric constant than isostatic pressing.	Optimization of dispersant dosage and pH is essential [28].
	CaCO_3_ (≥99.0%), CuO (≥99.0%) and TiO_2_ (≥99.0%) NaCl as molten salt	800 °C/2 h	Better dielectric performance than traditionally synthesized CCTO powder.	- [29]
Microwave flash combustion method	Ca(NO_3_)_2_·4H_2_O (99%, Merck),Cu(NO_3_)_2_·4H_2_O (99.5%, Merck), and TiO(NO_3_)_2_ oxidizers urea (CH_4_N_2_O) as fuel	800–900 °C/5 h	CCTO powder of particles size ranging from 50–70 nm was fabricated for the first using this technique.	- [30]
	Oxidizers (Ca(NO_3_)_2_4H_2_O, Cu (NO_3_)_2_ 4H_2_O and TiO(NO_3_)_2_), urea (CH_4_N_2_O)	900 °C/5 h	Nanocrystalline CCTO particles were formed.	Precautions are to be followed during combustion [31].

**Table 2:** The resistance of grains and grain boundaries of CaCu(3−x)CoxTi4O12 Ceramics (x = 0, 0.2, 0.4 and 0.6) [43].

Sample	R_g_ (Ω)	R_gb_ (Ω)
CaCu_3_Co_0_Ti_4_O_12_	43	5.5 × 10^5^
CaCu_2.8_Co_0.2_Ti_4_O_12_	52	9.5 × 10^6^
CaCu_2.6_Co_0.4_Ti_4_O_12_	11	3 × 10^7^
CaCu_2.4_Co_0.6_Ti_4_O_12_	77	3.2 × 10^8^

**Table 4:** Dielectric constant and loss of CCTO and Erbium dopants using microwave sintering at 1000 °C for 10min [77].

Frequency Hz	x = 0		x = 0.2		x = 0.5	
	ε_r_	tan δ	ε_r_	tan δ	ε_r_	tan δ
50	1730	0.16	5480	4.2	11,700	8.5
100	1633	0.15	3650	3.3	8310	6.1
1 k	1572	0.06	1850	0.9	3270	1.8
10 ck	1527	0.06	1280	0.3	1690	0.6
100 ck	1491	0.06	1010	0.1	1050	0.3
1 m	1462	0.17	790	0.3	762	0.2

## References

[1] Ramirez A., Subramanian M., Gardel M., Blumberg G., Li D., Vogt T., Shapiro S. (2000). Giant dielectric constant response in a copper-titanate. Solid State Commun..

[2] Subramanian M.A., Li D., Duan N., Reisner B.A., Sleight A.W. (2000). High Dielectric Constant in ACu_3_Ti_4_O_12_ and ACu_3_Ti_3_FeO_12_ Phases. J. Solid State Chem..

[3] Shao S.F., Zhang J.L., Zheng P., Zhong W.L., Wang C.L. (2006). Microstructure and electrical properties of CaCu_3_Ti_4_O_12_ ceramics. J. Appl. Phys..

[4] Ferrarelli M.C., Sinclair D.C., West A.R., Dabkowska H.A., Dabkowski A., Luke G.M. (2009). Comment on the origin(s) of the giant permittivity effect in CaCu_3_Ti_4_O_12_ single crystals and ceramics. J. Mater. Chem..

[5] Li Y., Liang P., Chao X., Yang Z. (2013). Preparation of CaCu_3_Ti_4_O_12_ ceramics with low dielectric loss and giant dielectric constant by the sol–gel technique. Ceram. Int..

[6] Singh L., Rai U., Mandal K. (2013). Dielectric properties of zinc doped nanocrystalline calcium copper titanate synthesized by different approach. Mater. Res. Bull..

[7] Bender B., Pan M.-J. (2005). The effect of processing on the giant dielectric properties of CaCu_3_Ti_4_O_12_. Mater. Sci. Eng. B.

[8] Xu C., Zhao X., Ren L., Sun J., Yang L., Guo J., Liao R. (2019). Enhanced electrical properties of CaCu_3_Ti_4_O_12_ ceramics by spark plasma sintering: Role of Zn and Al co-doping. J. Alloy. Compd..

[9] Löhnert R., Schmidt R., Töpfer J. (2015). Effect of sintering conditions on microstructure and dielectric properties of CaCu_3_Ti_4_O_12_ (CCTO) ceramics. J. Electroceramics.

[10] Huang X., Jiang Y., Wu K. (2015). CCTO Giant Dielectric Ceramic Prepared by Reaction Sintering. Procedia Eng..

[11] Late R., Rai H.M., Saxena S.K., Kumar R., Sagdeo P. (2015). Effect of hafnium substitution on the dielectric properties of CaCu_3_Ti_4_O_12_. AIP Conf. Proc..

[12] Wang Y., Ni L., Chen X.M. (2010). Effects of Nd-substitution on microstructures and dielectric characteristics of CaCu_3_Ti_4_O_12_ ceramics. J. Mater. Sci. Mater. Electron..

[13] Ren L., Yang L., Xu C., Zhao X., Liao R. (2018). Improvement of breakdown field and dielectric properties of CaCu_3_Ti_4_O_12_ ceramics by Bi and Al co-doping. J. Alloy. Compd..

[14] Chinnathambi M., Sakthisabarimoorthi A., Jose M., Robert R. (2021). Study of the Electrical and Dielectric behaviour of selenium doped CCTO ceramics prepared by a facile sol–gel route. Mater. Chem. Phys..

[15] Huang Y., Qiao Y., Li Y., He J., Zeng H. (2020). Zn-Doped Calcium Copper Titanate Synthesized via Rapid Laser Sintering of Sol–gel Derived Precursors. Nanomaterials.

[16] Samanta B., Kumar P., Nanda D. (2022). Effect of Al substitution and secondary CuO phase on dielectric response in microwave-processed CaCu_3_Ti_4−x_Al_x_O_12_ ceramics. J. Mater. Sci. Mater. Electron..

[17] Abdelal O.A., Hassan A.A., Ali M.E.S. (2014). Dielectric Properties of Calcium Copper Titanates (CaCu_3_Ti_4_O_12_) Synthesized by Solid State Reaction. Int. J. Adv. Res. Chem. Sci..

[18] Boonlakhorn J., Nijpanich S., Thongbai P., Srepusharawoot P. (2022). High dielectric permittivity and dielectric relaxation behavior in a Y2/3Cu_3_Ti_4_O_12_ ceramic prepared by a modified Sol−Gel route. Ceram. Int..

[19] Aliabadi T.N., Alizadeh P. (2019). Microstructure and dielectric properties of CCTO glass-ceramic prepared by the melt-quenching method. Ceram. Int..

[20] Mao P., Wang J., Zhang L., Liu S., Zhao Y., Sun Q. (2019). Rapid fabrication and improved electrical properties of CaCu_3_Ti_4_O_12_ ceramics by sol–gel and spark plasma sintering techniques. J. Mater. Sci. Mater. Electron..

[21] Riquet G., Marinel S., Breard Y., Harnois C., Pautrat A. (2018). Direct and hybrid microwave solid state synthesis of CaCu_3_Ti_4_O_12_ ceramic: Microstructures and dielectric properties. Ceram. Int..

[22] Thomas P., Sathapathy L.N., Dwarakanath K., Varma K.B.R. (2007). Microwave synthesis and sintering characteristics of CaCu_3_Ti_4_O_12_. Bull. Mater. Sci..

[23] Yu H., Liu H., Luo D., Cao M. (2008). Microwave synthesis of high dielectric constant CaCu_3_Ti_4_O_12_. J. Mater. Process. Technol..

[24] Ouyang X., Cao P., Huang S., Zhang W., Huang Z., Gao W. (2015). Microwave-Assisted Synthesis of High Dielectric Constant CaCu_3_Ti_4_O_12_ from Sol–Gel Precursor. J. Electron. Mater..

[25] Chandrasekhar M., Kumar P. (2017). Microwave Assisted Sol–gel Synthesis of High Dielectric Constant CCTO and BFN Ceramics for MLC Applica-tions. Processing Appl. Ceram..

[26] Chen K.P., He Y., Liu D.Y., De Liu Z. (2008). Molten Salt Synthesis of CaCu_3_Ti_4_O_12_. Key Eng. Mater..

[27] Prakash B.S., Varma K.B.R. (2008). Molten salt synthesis of nanocrystalline phase of high dielectric constant material CaCu_3_Ti_4_O_12_. J. Nanosci. Nanotechnol..

[28] Wan W., Liu C., Sun H., Luo Z., Yuan W.-X., Wu H., Qiu T. (2015). Low-toxic gelcasting of giant dielectric-constant CaCu_3_Ti_4_O_12_ ceramics from the molten salt powder. J. Eur. Ceram. Soc..

[29] Wan W., Yang J., Yuan W.-X., Zhao X., Liu C., Qiu T. (2015). Preparation of Giant Dielectric CaCu_3_Ti_4_O_12_Ceramics via the Molten Salt Method from NaCl Flux. Int. J. Appl. Ceram. Technol..

[30] Kumar R., Zulfequar M., Sharma L., Singh V.N., Senguttuvan T.D. (2015). Growth of Nanocrystalline CaCu_3_Ti_4_O_12_ Ceramic by the Microwave Flash Combustion Method: Structural and Impedance Spectroscopic Studies. Cryst. Growth Des..

[31] Kumar R., Zulfequar M., Senguttuvan T.D. (2015). Dielectric properties of microwave flash combustion derived and spark plasma sintered CaCu_3_Ti_4_O_12_ ceramic: Role of reduction in grain boundary activation energy. J. Mater. Sci. Mater. Electron..

[32] Supriya D.M., Rajani M.R., Phani A.R., Naveen C.V.S., Ravishankar R. (2017). Synthesis of CCTO and Doped CCTO Nanopowders and its Applications in the Field of Electronics. Mater. Today Proc..

[33] Zhao J., Chen M., Tan Q. (2021). Embedding nanostructure and colossal permittivity of TiO2-covered CCTO perovskite materials by a hydrothermal route. J. Alloy. Compd..

[34] Wang H., Li S., He J., Lin C. (2014). Dielectric properties of CaCu_3_Ti_4_O_12_ ceramics: Effect of high purity nanometric powders. J. Mater. Sci. Mater. Electron..

[35] Fiorenza P., Raineri V., Ebbinghaus S.G., Nigro R.L. (2011). CaCu_3_Ti_4_O_12_ single crystals: Insights on growth and nanoscopic investigation. CrystEngComm.

[36] Ramadan R.M., Labeeb A.M., Ward A., Ibrahim A.M.H. (2020). New approach for synthesis of nano-sized CaCu_3_Ti_4_O_12_ powder by economic and innovative method. J. Mater. Sci. Mater. Electron..

[37] Babu S., Govindan A. (2014). Dielectric Properties of CaCu_3_Ti_4_O_12_ (CCTO) Prepared by Modified Solid State Reaction Method. Int. Rev. Appl. Eng. Res..

[38] Zhao J., Zhao H., Zhu Z. (2019). Influence of sintering conditions and CuO loss on dielectric properties of CaCu_3_Ti_4_O_12_ ceramics. Mater. Res. Bull..

[39] Riquet G., Marinel S., Bréard Y., Harnois C. (2019). Sintering mechanism and grain growth in CaCu_3_Ti_4_O_12_ ceramics. Ceram. Int..

[40] Wang X.W., Jia P.B., Zhang B.H., Sun L.Y., Liu Q.B. (2016). Calcining temperature dependence on structure and dielectric properties of CaCu_3_Ti_4_O_12_ ceramics. J. Mater. Sci. Mater. Electron..

[41] Senda S., Rhouma S., Torkani E., Megriche A., Autret C. (2017). Effect of nickel substitution on electrical and microstructural properties of CaCu_3_Ti_4_O_12_ ceramic. J. Alloy. Compd..

[42] Zhuk N., Sekushin N., Krzhizhanovskaya M., Belyy V., Korolev R. (2021). Electrical properties of Ni-doped CaCu_3_Ti_4_O_12_ ceramics. Solid State Ionics.

[43] Kafi Z., Kompany A., Arabi H., Zak A.K. (2017). The effect of cobalt-doping on microstructure and dielectric properties of CaCu_3_Ti_4_O_12_ ceramics. J. Alloy. Compd..

[44] Yanchevskii O., V’Yunov O., Belous A., Kovalenko L. (2021). Dielectric properties of CaCu_3_Ti_4_O_12_ ceramics doped with aluminium and fluorine. J. Alloy. Compd..

[45] Boonlakhorn J., Srepusharawoot P., Thongbai P. (2019). Distinct roles between complex defect clusters and insulating grain boundary on dielectric loss behaviors of (In^3+^/Ta^5+^) co-doped CaCu_3_Ti_4_O_12_ ceramics. Results Phys..

[46] Boonlakhorn J., Chanlek N., Thongbai P., Srepusharawoot P. (2020). Strongly Enhanced Dielectric Response and Structural Investigation of (Sr^2+^, Ge^4+^) Co-Doped CCTO Ceramics. J. Phys. Chem. C.

[47] Jumpatam J., Putasaeng B., Chanlek N., Manyam J., Srepusharawoot P., Krongsuk S., Thongbai P. (2021). Influence of Sn and F dopants on giant dielectric response and Schottky potential barrier at grain boundaries of CCTO ceramics. Ceram. Int..

[48] Liu J., Lu D.-Y., Yu X.-Y., Liu Q., Tao Q., Change H., Zhu P.-W. (2016). Dielectric Properties of Eu-Doped CaCu_3_Ti_4_O_12_ with Different Compensation Mechanisms. Acta Met. Sin..

[49] Liu L., Fang L., Huang Y., Li Y., Shi D., Zheng S., Wu S., Hu C. (2011). Dielectric and nonlinear current–voltage characteristics of rare–earth doped CaCu_3_Ti_4_O_12_ ceramics. J. Appl. Phys..

[50] Xue R., Chen Z., Dai H., Liu D., Li T., Zhao G. (2015). Effects of rare earth ionic doping on microstructures and electrical properties of CaCu_3_Ti_4_O_12_ ceramics. Mater. Res. Bull..

[51] Mu C., Zhang H., Liu Y., Song Y., Liu P. (2010). Rare earth doped CaCu_3_Ti_4_O_12_ electronic ceramics for high frequency applications. J. Rare Earths.

[52] Kashyap R., Thakur O., Tandon R. (2012). Study of structural, dielectric and electrical conduction behaviour of Gd substituted CaCu_3_Ti_4_O_12_ ceramics. Ceram. Int..

[53] Mamedov V. (2002). Spark plasma sintering as advanced PM sintering method. Powder Met..

[54] Cavaliere P., Sadeghi B., Shabani A., Cavaliere P. (2019). Spark Plasma Sintering: Process Fundamentals. Spark Plasma Sintering of Materials: Advances in Processing and Applications.

[55] Suárez M., Fernández A., Menéndez J., Torrecillas R., Kessel H.U., Hennicke J., Kirchner R., Kessel T. (2013). Challenges and Opportunities for Spark Plasma Sintering: A Key Technology for a New Generation of Materials. Sinter. Appl..

[56] Munir Z.A., Anselmi-Tamburini U., Ohyanagi M. (2006). The effect of electric field and pressure on the synthesis and consolidation of materials: A review of the spark plasma sintering method. J. Mater. Sci..

[57] Munir Z.A., Ohyanagi M. (2020). Perspectives on the spark plasma sintering process. J. Mater. Sci..

[58] de Carvalho E., Bertolete M., Machado I.F., Muccillo E. (2012). Effect of the Dwell Temperature on Spark Plasma Sintered CaCu_3_Ti_4_O_12_. Mater. Sci. Forum.

[59] Ruan X.F., Yang Z., Zhang Y., Shi J., Xiong R. (2013). The Synthesis of CaCu_3_Ti_4_O_12_ High Dielectric Ceramics by a Spark Plasma Sintering Method. Key Eng. Mater..

[60] Ahmad M.M., Yamada K. (2014). Grain size effect on the giant dielectric constant of CaCu_3_Ti_4_O_12_ nanoceramics prepared by mechanosynthesis and spark plasma sintering. J. Appl. Phys..

[61] Lin H., He X., Gong Y., Pang D., Yi Z. (2018). Tuning the nonlinear current-voltage behavior of CaCu_3_Ti_4_O_12_ ceramics by spark plasma sintering. Ceram. Int..

[62] Li T., Sun Y., Dai H., Liu D., Chen J., Xue R., Chen Z. (2020). Influence of spark plasma sintering temperature on the microstructures, dielectric and I–V properties of CaCu_3_Ti_4_O_12_ ceramics. J. Alloy. Compd..

[63] Kotb H.M., Ahmad M.M., Aldabal S., Alshoaibi A., Aljaafari A. (2019). Structural and dielectric behavior of Al-substituted CaCu_3_Ti_4_O_12_ ceramics with giant dielectric constant by spark plasma sintering. J. Mater. Sci. Mater. Electron..

[64] Chatterjee A., Basak T., Ayappa K.G. (1998). Analysis of microwave sintering of ceramics. AIChE J..

[65] Das S., Mukhopadhyay A.K., Datta S., Basu D. (2009). Prospects of microwave processing: An overview. Bull. Mater. Sci..

[66] Agrawal D.K. (1998). Microwave processing of ceramics. Curr. Opin. Solid State Mater. Sci..

[67] Rybakov K.I., Olevsky E.A., Krikun E.V. (2013). Microwave Sintering: Fundamentals and Modeling. J. Am. Ceram. Soc..

[68] Boch P., Lequeux N. (1997). Do microwaves increase the sinterability of ceramics?. Solid State Ionics.

[69] Borrell A., Salvador M.D., Malin Liu M. (2018). Advanced Ceramic Materials Sintered by Microwave Technology. Sintering Technology—Method and Application.

[70] Oghbaei M., Mirzaee O. (2010). Microwave versus conventional sintering: A review of fundamentals, advantages and applications. J. Alloy. Compd..

[71] Sulaiman M.A. (2020). Effect of Calcination Time on the Microstructure and Dielectric Properties of Ca-Cu_3_Ti_4_O_12_ Using Enhanced Microwave Processing. Malays. J. Microsc..

[72] Hutagalung S.D., Ibrahim M.I.M., Ahmad Z.A. (2008). Microwave assisted sintering of CaCu_3_Ti_4_O_12_. Ceram. Int..

[73] A Ramírez M., Bueno P.R., Longo E., A Varela J. (2008). Conventional and microwave sintering of CaCu_3_Ti_4_O_12_/CaTiO_3_ceramic composites: Non-ohmic and dielectric properties. J. Phys. D Appl. Phys..

[74] Kumar R., Zulfequar M., Singh V., Tawale J., Senguttuvan T. (2012). Microwave sintering of dielectric CaCu_3_Ti_4_O_12_: An interfacial conductance and dipole relaxation effect. J. Alloy. Compd..

[75] Jesurani S., Kanagesan S., Hashim M., Ismail I., Sabbaghizadeh R. (2014). Influence of Er on microstructural and dielectric properties of CaCu_3_Ti_4_O_12_. J. Mater. Sci. Mater. Electron..

[76] Evangeline T.G., Annamalai A.R. (2022). Influence of heating modes on the microstructural and dielectric properties of calcium copper titanium oxide (CaCu_3_Ti_4_O_12_/CCTO) using conventional and microwave sintering. J. Mater. Sci. Mater. Electron..

[77] Evangeline T.G., Annamalai A.R. (2022). Dielectric properties of conventional and microwave-sintered Lanthanum doped CaCu_3_Ti_4_O_12_ ceramics for high-frequency applications. Ceram. Int..

[78] Kawrani S., Boulos M., Cornu D., Bechelany M. (2019). From Synthesis to Applications: Copper Calcium Titanate (CCTO) and its Magnetic and Photocatalytic Properties. ChemistryOpen.

[79] Ahmadipour M., Ain M.F., Ahmad Z.A. (2016). A Short Review on Copper Calcium Titanate (CCTO) Electroceramic: Synthesis, Dielectric Properties, Film Deposition, and Sensing Application. Nano-Micro Lett..

[80] Qu Y., Wu Y., Fan G., Xie P., Liu Y., Zhang Z., Xin J., Jiang Q., Sun K., Fan R. (2020). Tunable radio-frequency negative permittivity of Carbon/CaCu_3_Ti_4_O_12_ metacomposites. J. Alloy. Compd..

[81] Ma R., Cheng C., Qu Y., Fan R. (2020). Tailorable negative permittivity of graphene-carbon nanotube/copper calcium titanate metacomposites. Ceram. Int..

[82] Singh J.P., Gautam S., Kumar P., Tripathi A., Chen J.-M., Chae K.H., Asokan K. (2013). Correlation between the dielectric properties and local electronic structure of copper doped calcium titanate. J. Alloy. Compd..

[83] Mohammed J., Carol T.T.C., Hafeez H., Adamu B., Wudil Y., Takai Z., Godara S.K., Srivastava A. (2018). Tuning the dielectric and optical properties of Pr Co–substituted calcium copper titanate for electronics applications. J. Phys. Chem. Solids.

[84] Wang J., Chao X., Li G., Feng L., Zhao K. (2016). Fabrication and enhanced characterization of copper powder filled copper calcium titanate/poly(vinylidene difluoride) composite. J. Mater. Sci. Mater. Electron..

[85] Wang S., He X., Chen Q., Chen Y., He W., Zhou G., Zhang H., Jin X., Su X. (2018). Graphene-coated copper calcium titanate to improve dielectric performance of PPO-based composite. Mater. Lett..

[86] Li X., Wang J., Chen H., Xiong C., Shi Z., Yang Q. (2021). Flexible dielectric nanocomposite films based on chitin/boron nitride/copper calcium titanate with high energy density. Compos. Part. A Appl. Sci. Manuf..

[87] Ding B., Lin J., Wan F., Qu Y., Wu J., Sun K., Fan R. (2020). Communication—Tunable Epsilon-Negative Property of Nickel/Copper Calcium Titanate Cermets. ECS J. Solid State Sci. Technol..

[88] Luangchuang P., Chueangchayaphan N., Sulaiman M.A., Chueangchayaphan W. (2020). Evaluation of cure characteristic, physico-mechanical, and dielectric properties of calcium copper titanate filled acrylonitrile-butadiene rubber composites: Effect of calcium copper titanate loading. J. Appl. Polym. Sci..

[89] Chattopadhyay A., Nayak J. (2022). Improvement of humidity sensing performance and dielectric response through pH variation in CaCu_3_Ti_4_O_12_ ceramics. Sensors Actuators A Phys..

[90] Tomchenko A.A., Harmer G.P., Marquis B.T., Allen J.W. (2003). Semiconducting metal oxide sensor array for the selective detection of combustion gases. Sensors Actuators B Chem..

[91] Hilder M., Winther-Jensen B., Clark N. (2009). Paper-based, printed zinc–air battery. J. Power Sources.

[92] Hu L., Choi J.W., Yang Y., Jeong S., La Mantia F., Cui L.-F., Cui Y. (2009). Highly conductive paper for energy-storage devices. Proc. Natl. Acad. Sci. USA.

[93] Liu H., Crooks R.M. (2012). Paper-Based Electrochemical Sensing Platform with Integral Battery and Electrochromic Read-Out. Anal. Chem..

[94] Zhong Q., Zhong J., Hu B., Hu Q., Zhou J., Wang Z.L. (2013). A paper-based nanogenerator as a power source and active sensor. Energy Environ. Sci..

[95] Bu Y., Gwon O., Nam G., Jang H., Kim S., Zhong Q., Cho J., Kim G. (2017). A Highly Efficient and Robust Cation Ordered Perovskite Oxide as a Bifunctional Catalyst for Rechargeable Zinc–air Batteries. ACS Nano.

[96] Fu J., Cano Z., Park M.G., Yu A., Fowler M., Chen Z. (2016). Electrically Rechargeable Zinc–air Batteries: Progress, Challenges, and Perspectives. Adv. Mater..

[97] Li Y., Dai H. (2014). Recent advances in zinc–air batteries. Chem. Soc. Rev..

[98] Park H.-S., Seo E., Yang J., Lee Y., Kim B.-S., Song H.-K. (2017). Bifunctional hydrous RuO_2_ nanocluster electrocatalyst embedded in carbon matrix for efficient and durable operation of rechargeable zinc–air batteries. Sci. Rep..

[99] Bhardwaj U., Sharma A., Gupta V., Batoo K.M., Hussain S., Kushwaha H.S. (2022). High energy storage capabilities of CaCu_3_Ti_4_O_12_ for paper-based zinc–air battery. Sci. Rep..

[100] Pandey R.K., Stapleton W.A., Tate J., Bandyopadhyay A.K., Sutanto I., Sprissler S., Lin S. (2013). Applications of CCTO supercapacitor in energy storage and electronics. AIP Adv..

[101] Kaur S., Kumar A., Sharma A.L., Singh D.P. (2019). Dielectric and energy storage behavior of CaCu_3_Ti_4_O_12_ nanoparticles for capacitor application. Ceram. Int..

[102] Kushwaha H.S., A Madhar N., Ilahi B., Thomas P., Halder A., Vaish R. (2016). Efficient Solar Energy Conversion Using CaCu_3_Ti_4_O_12_ Photoanode for Photocatalysis and Photoelectrocatalysis. Sci. Rep..

[103] Kawrani S., Boulos M., Bekheet M.F., Viter R., Nada A., Riedel W., Roualdes S., Cornu D., Bechelany M. (2020). Segregation of copper oxide on calcium copper titanate surface induced by Graphene Oxide for Water splitting applications. Appl. Surf. Sci..

[104] Irshad M., Ain Q.T., Zaman M., Aslam M.Z., Kousar N., Asim M., Rafique M., Siraj K., Tabish A.N., Usman M. (2022). Photocatalysis and perovskite oxide-based materials: A remedy for a clean and sustainable future. RSC Adv..

[105] Clark J.H., Dyer M.S., Palgrave R.G., Ireland C.P., Darwent J.R., Claridge J.B., Rosseinsky M.J. (2010). Visible Light Photo-oxidation of Model Pollutants Using CaCu_3_Ti_4_O_12_: An Experimental and Theoretical Study of Optical Properties, Electronic Structure, and Selectivity. J. Am. Chem. Soc..

[106] Wang M., Liu J., Xu C., Feng L. (2022). Sonocatalysis and sono-photocatalysis in CaCu_3_Ti_4_O_12_ ceramics. Ceram. Int..

[107] Saqib N.U., Shah I., Adnan R. (2022). An Emerging Photocatalyst for Wastewater Remediation: A Mini-Review on CaCu_3_Ti_4_O_12_ Photoca-talysis. Environ. Sci. Pollut. Res..

